# 1,3-Dibenzyl-2-methyl­benzimidazolium chloride

**DOI:** 10.1107/S1600536810002588

**Published:** 2010-01-27

**Authors:** Hamid Ennajih, Rachid Bouhfid, Hafid Zouihri, El Mokhtar Essassi, Seik Weng Ng

**Affiliations:** a Institute of Nanomaterials and Nanotechnology, Avenue de l’Armée Royale, Madinat El Irfane, 10100 Rabat, Morocco; b CNRST Division of UATRS Angle Allal Fassi/FAR, BP 8027 Hay Riad, 10000 Rabat, Morocco; c Laboratoire de Chimie Organique Hétérocyclique, Pôle de Compétences Pharmacochimie, Université Mohammed V-Agdal, BP 1014 Avenue Ibn Batout, Rabat, Morocco; d Department of Chemistry, University of Malaya, 50603 Kuala Lumpur, Malaysia

## Abstract

The cation of the title salt, C_22_H_21_N_2_^+^·Cl^−^, contains a planar benzimidazolium unit (r.m.s. deviation = 0.02 Å); the phenyl rings of the benzyl substituents form dihedral angles of 68.2 (1) and 79.7 (1)° with the plane of the benzimidazolium fragment.

## Related literature

For the crystal structure of the monohydrated salt, see: Jian *et al.* (2003 ▶).
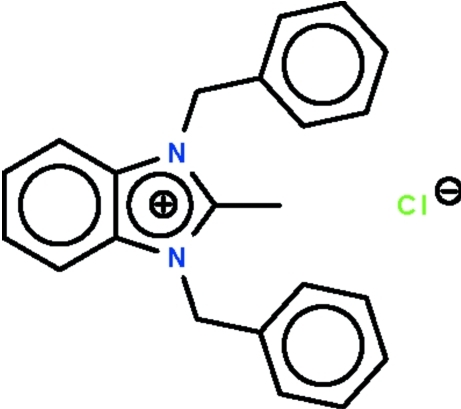


## Experimental

### 

#### Crystal data

C_22_H_21_N_2_^+^·Cl^−^
*M* *_r_* = 348.86
Triclinic, 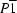

*a* = 9.2539 (2) Å
*b* = 9.4677 (2) Å
*c* = 12.0984 (3) Å
α = 72.139 (1)°
β = 81.376 (1)°
γ = 64.605 (1)°
*V* = 911.20 (4) Å^3^
*Z* = 2
Mo *K*α radiation
μ = 0.22 mm^−1^
*T* = 293 K
0.30 × 0.30 × 0.30 mm

#### Data collection

Bruker APEXII diffractometer
Absorption correction: multi-scan (*SADABS*; Sheldrick, 1996 ▶) *T*_min_ = 0.938, *T*_max_ = 0.938
24459 measured reflections
4175 independent reflections
3336 reflections with *I* > 2σ(*I*)
*R*_int_ = 0.028

#### Refinement

*R*[*F* ^2^ > 2σ(*F* ^2^)] = 0.038
*wR*(*F* ^2^) = 0.124
*S* = 1.08
4175 reflections
227 parameters
H-atom parameters constrained
Δρ_max_ = 0.23 e Å^−3^
Δρ_min_ = −0.23 e Å^−3^

### 

Data collection: *APEX2* (Bruker, 2005 ▶); cell refinement: *SAINT* (Bruker, 2005 ▶); data reduction: *SAINT*; program(s) used to solve structure: *SHELXS97* (Sheldrick, 2008 ▶); program(s) used to refine structure: *SHELXL97* (Sheldrick, 2008 ▶); molecular graphics: *X-SEED* (Barbour, 2001 ▶); software used to prepare material for publication: *publCIF* (Westrip, 2010 ▶).

## Supplementary Material

Crystal structure: contains datablocks global, I. DOI: 10.1107/S1600536810002588/bt5179sup1.cif

Structure factors: contains datablocks I. DOI: 10.1107/S1600536810002588/bt5179Isup2.hkl

Additional supplementary materials:  crystallographic information; 3D view; checkCIF report

## Figures and Tables

**Fig. 1.**
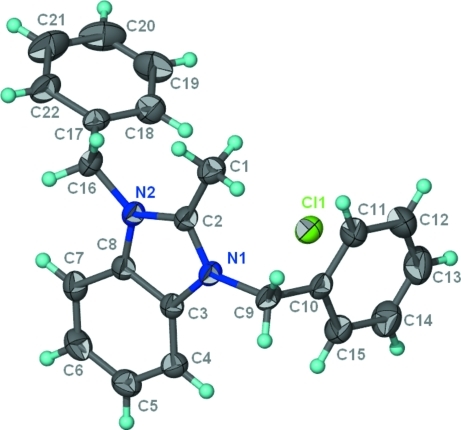
Anisotropic displacement ellipsoid plot (Barbour, 2001) of the title compound at the 50% probability level; hydrogen atoms are drawn as spheres of an arbitrary radius.

## supplementary crystallographic information

### Experimental

To a solution of 2-methylbenzimidazole (1 g, 7.57 mmol) in DMF (20 ml) was added benzyl chloride (2,66 ml, 22.7 mmol), potassium carbonate (1.25 g, 9.08 mmol) and a catalytic amount of tetra-*n*-butylammonium bromide. The mixture was stirred for 24 h. The solution was filtered and the solvent removed under reduced pressure. The residue was recrystallized from ethanol to afford 1,3-dibenzyl-2-methyl-benzimidazolium chloride as colorless crystals.

### Refinement

H-atoms were placed in calculated positions (C—H 0.93–0.97 Å) and were included in the refinement in the riding model approximation, with *U*(H) set to 1.2–1.5*U*(C).

### Crystal data

**Table:**

C_22_H_21_N_2_^+^·Cl^−^	*Z* = 2
*M**_r_* = 348.86	*F*(000) = 368
Triclinic, *P*1	*D*_x_ = 1.271 Mg m^−^^3^
Hall symbol: -P 1	Mo *K*α radiation, λ = 0.71073 Å
*a* = 9.2539 (2) Å	Cell parameters from 8258 reflections
*b* = 9.4677 (2) Å	θ = 2.5–29.1°
*c* = 12.0984 (3) Å	µ = 0.22 mm^−^^1^
α = 72.139 (1)°	*T* = 293 K
β = 81.376 (1)°	Block, colorless
γ = 64.605 (1)°	0.30 × 0.30 × 0.30 mm
*V* = 911.20 (4) Å^3^	

### Data collection

**Table:**

Bruker APEXII diffractometer	4175 independent reflections
Radiation source: fine-focus sealed tube	3336 reflections with *I* > 2σ(*I*)
graphite	*R*_int_ = 0.028
φ and ω scans	θ_max_ = 27.5°, θ_min_ = 1.8°
Absorption correction: multi-scan (*SADABS*; Sheldrick, 1996)	*h* = −12→12
*T*_min_ = 0.938, *T*_max_ = 0.938	*k* = −12→12
24459 measured reflections	*l* = −15→15

### Refinement

**Table:**

Refinement on *F*^2^	Primary atom site location: structure-invariant direct methods
Least-squares matrix: full	Secondary atom site location: difference Fourier map
*R*[*F*^2^ > 2σ(*F*^2^)] = 0.038	Hydrogen site location: inferred from neighbouring sites
*wR*(*F*^2^) = 0.124	H-atom parameters constrained
*S* = 1.08	*w* = 1/[σ^2^(*F*_o_^2^) + (0.0687*P*)^2^ + 0.1373*P*] where *P* = (*F*_o_^2^ + 2*F*_c_^2^)/3
4175 reflections	(Δ/σ)_max_ = 0.001
227 parameters	Δρ_max_ = 0.23 e Å^−^^3^
0 restraints	Δρ_min_ = −0.23 e Å^−^^3^

### Fractional atomic coordinates and isotropic or equivalent isotropic displacement parameters (Å^2^)

**Table:**

	*x*	*y*	*z*	*U*_iso_*/*U*_eq_	
Cl1	0.29429 (4)	0.33126 (4)	0.00802 (3)	0.04507 (14)	
N1	0.77776 (14)	0.02156 (14)	0.14627 (10)	0.0356 (3)	
N2	0.91155 (14)	0.15323 (14)	0.16783 (10)	0.0352 (3)	
C1	0.61460 (19)	0.30695 (19)	0.16123 (16)	0.0498 (4)	
H1A	0.5794	0.3046	0.2403	0.075*	
H1B	0.5340	0.3064	0.1200	0.075*	
H1C	0.6328	0.4036	0.1249	0.075*	
C2	0.76494 (17)	0.16248 (17)	0.15880 (12)	0.0360 (3)	
C3	0.93932 (17)	−0.08252 (17)	0.14577 (12)	0.0349 (3)	
C4	1.0170 (2)	−0.23710 (18)	0.12901 (14)	0.0434 (4)	
H4	0.9608	−0.2928	0.1179	0.052*	
C5	1.1821 (2)	−0.3032 (2)	0.12974 (15)	0.0515 (4)	
H5	1.2387	−0.4067	0.1194	0.062*	
C6	1.2669 (2)	−0.2194 (2)	0.14549 (16)	0.0541 (4)	
H6	1.3781	−0.2688	0.1458	0.065*	
C7	1.18996 (19)	−0.0654 (2)	0.16059 (14)	0.0454 (4)	
H7	1.2462	−0.0092	0.1704	0.054*	
C8	1.02401 (17)	0.00110 (17)	0.16028 (12)	0.0350 (3)	
C9	0.64487 (18)	−0.02047 (18)	0.13464 (14)	0.0416 (3)	
H9	0.6788	−0.0902	0.0830	0.050*	
H9B	0.5545	0.0780	0.1000	0.050*	
C10	0.59317 (18)	−0.10561 (18)	0.25015 (14)	0.0411 (3)	
C11	0.4779 (2)	−0.0170 (2)	0.3180 (2)	0.0646 (5)	
H11	0.4329	0.0959	0.2926	0.078*	
C12	0.4288 (3)	−0.0946 (3)	0.4233 (2)	0.0821 (7)	
H12	0.3506	−0.0341	0.4682	0.099*	
C13	0.4951 (3)	−0.2606 (3)	0.4616 (2)	0.0750 (6)	
H13	0.4628	−0.3126	0.5329	0.090*	
C14	0.6089 (3)	−0.3505 (3)	0.3951 (2)	0.0668 (5)	
H14	0.6539	−0.4633	0.4212	0.080*	
C15	0.6566 (2)	−0.2729 (2)	0.28935 (17)	0.0526 (4)	
H15	0.7325	−0.3343	0.2438	0.063*	
C16	0.95196 (19)	0.28401 (18)	0.17480 (13)	0.0405 (3)	
H16	0.8649	0.3876	0.1434	0.049*	
H16B	1.0466	0.2809	0.1268	0.049*	
C17	0.98260 (18)	0.27339 (17)	0.29640 (13)	0.0380 (3)	
C18	0.9089 (2)	0.2087 (2)	0.39402 (15)	0.0533 (4)	
H18	0.8402	0.1638	0.3866	0.064*	
C19	0.9367 (3)	0.2104 (3)	0.50305 (17)	0.0700 (6)	
H19	0.8861	0.1670	0.5682	0.084*	
C20	1.0372 (3)	0.2749 (3)	0.5152 (2)	0.0813 (7)	
H20	1.0558	0.2754	0.5885	0.098*	
C21	1.1109 (3)	0.3392 (3)	0.4191 (2)	0.0821 (7)	
H21	1.1794	0.3838	0.4274	0.099*	
C22	1.0846 (2)	0.3384 (2)	0.31030 (18)	0.0574 (5)	
H22	1.1359	0.3820	0.2457	0.069*	

### Atomic displacement parameters (Å^2^)

**Table:**

	*U*^11^	*U*^22^	*U*^33^	*U*^12^	*U*^13^	*U*^23^
Cl1	0.0415 (2)	0.0398 (2)	0.0544 (2)	−0.01604 (16)	−0.00553 (16)	−0.01205 (16)
N1	0.0346 (6)	0.0361 (6)	0.0407 (6)	−0.0176 (5)	−0.0027 (5)	−0.0109 (5)
N2	0.0371 (6)	0.0385 (6)	0.0354 (6)	−0.0199 (5)	−0.0011 (5)	−0.0107 (5)
C1	0.0415 (9)	0.0420 (8)	0.0686 (11)	−0.0158 (7)	−0.0026 (8)	−0.0196 (8)
C2	0.0385 (7)	0.0371 (7)	0.0366 (7)	−0.0191 (6)	−0.0017 (6)	−0.0098 (6)
C3	0.0364 (7)	0.0378 (7)	0.0309 (7)	−0.0168 (6)	−0.0005 (5)	−0.0076 (5)
C4	0.0486 (9)	0.0399 (8)	0.0437 (8)	−0.0196 (7)	0.0023 (7)	−0.0133 (6)
C5	0.0488 (9)	0.0421 (8)	0.0559 (10)	−0.0122 (7)	0.0056 (8)	−0.0155 (7)
C6	0.0362 (8)	0.0562 (10)	0.0601 (10)	−0.0120 (7)	0.0031 (7)	−0.0147 (8)
C7	0.0374 (8)	0.0555 (9)	0.0460 (9)	−0.0224 (7)	−0.0002 (6)	−0.0125 (7)
C8	0.0372 (7)	0.0397 (7)	0.0303 (7)	−0.0181 (6)	−0.0002 (5)	−0.0093 (5)
C9	0.0385 (8)	0.0411 (7)	0.0527 (9)	−0.0201 (6)	−0.0082 (7)	−0.0139 (7)
C10	0.0343 (7)	0.0437 (8)	0.0543 (9)	−0.0211 (6)	−0.0024 (6)	−0.0171 (7)
C11	0.0597 (11)	0.0542 (10)	0.0887 (15)	−0.0312 (9)	0.0230 (10)	−0.0311 (10)
C12	0.0870 (16)	0.0893 (16)	0.0946 (17)	−0.0553 (14)	0.0443 (14)	−0.0511 (14)
C13	0.0851 (16)	0.0911 (16)	0.0633 (13)	−0.0583 (14)	0.0143 (11)	−0.0146 (11)
C14	0.0671 (12)	0.0537 (10)	0.0756 (13)	−0.0304 (10)	−0.0002 (10)	−0.0043 (10)
C15	0.0498 (10)	0.0451 (9)	0.0647 (11)	−0.0213 (8)	0.0049 (8)	−0.0171 (8)
C16	0.0460 (8)	0.0400 (7)	0.0427 (8)	−0.0255 (7)	−0.0023 (6)	−0.0084 (6)
C17	0.0377 (7)	0.0307 (6)	0.0463 (8)	−0.0109 (6)	−0.0046 (6)	−0.0141 (6)
C18	0.0619 (11)	0.0563 (10)	0.0450 (9)	−0.0277 (9)	−0.0002 (8)	−0.0134 (8)
C19	0.0869 (15)	0.0691 (13)	0.0454 (10)	−0.0223 (11)	−0.0005 (10)	−0.0183 (9)
C20	0.1008 (18)	0.0796 (15)	0.0648 (14)	−0.0186 (13)	−0.0261 (13)	−0.0366 (12)
C21	0.0938 (17)	0.0875 (16)	0.0920 (17)	−0.0434 (14)	−0.0242 (14)	−0.0406 (14)
C22	0.0606 (11)	0.0575 (10)	0.0691 (12)	−0.0315 (9)	−0.0070 (9)	−0.0236 (9)

### Geometric parameters (Å, °)

**Table:**

N1—C2	1.3416 (18)	C10—C11	1.382 (2)
N1—C3	1.3941 (18)	C11—C12	1.381 (3)
N1—C9	1.4821 (18)	C11—H11	0.9300
N2—C2	1.3406 (18)	C12—C13	1.368 (3)
N2—C8	1.3902 (18)	C12—H12	0.9300
N2—C16	1.4664 (18)	C13—C14	1.370 (3)
C1—C2	1.479 (2)	C13—H13	0.9300
C1—H1A	0.9600	C14—C15	1.380 (3)
C1—H1B	0.9600	C14—H14	0.9300
C1—H1C	0.9600	C15—H15	0.9300
C3—C8	1.389 (2)	C16—C17	1.505 (2)
C3—C4	1.391 (2)	C16—H16	0.9700
C4—C5	1.381 (2)	C16—H16B	0.9700
C4—H4	0.9300	C17—C22	1.381 (2)
C5—C6	1.397 (3)	C17—C18	1.381 (2)
C5—H5	0.9300	C18—C19	1.387 (3)
C6—C7	1.378 (2)	C18—H18	0.9300
C6—H6	0.9300	C19—C20	1.358 (4)
C7—C8	1.388 (2)	C19—H19	0.9300
C7—H7	0.9300	C20—C21	1.368 (4)
C9—C10	1.507 (2)	C20—H20	0.9300
C9—H9	0.9700	C21—C22	1.377 (3)
C9—H9B	0.9700	C21—H21	0.9300
C10—C15	1.380 (2)	C22—H22	0.9300
			
C2—N1—C3	108.77 (12)	C11—C10—C9	120.38 (15)
C2—N1—C9	126.81 (12)	C10—C11—C12	120.55 (19)
C3—N1—C9	124.43 (12)	C10—C11—H11	119.7
C2—N2—C8	108.89 (12)	C12—C11—H11	119.7
C2—N2—C16	126.78 (13)	C13—C12—C11	120.0 (2)
C8—N2—C16	124.15 (12)	C13—C12—H12	120.0
C2—C1—H1A	109.5	C11—C12—H12	120.0
C2—C1—H1B	109.5	C14—C13—C12	120.2 (2)
H1A—C1—H1B	109.5	C14—C13—H13	119.9
C2—C1—H1C	109.5	C12—C13—H13	119.9
H1A—C1—H1C	109.5	C13—C14—C15	119.71 (19)
H1B—C1—H1C	109.5	C13—C14—H14	120.1
N2—C2—N1	109.12 (13)	C15—C14—H14	120.1
N2—C2—C1	124.66 (13)	C10—C15—C14	120.95 (18)
N1—C2—C1	126.21 (13)	C10—C15—H15	119.5
C8—C3—C4	121.57 (14)	C14—C15—H15	119.5
C8—C3—N1	106.52 (12)	N2—C16—C17	113.56 (12)
C4—C3—N1	131.83 (14)	N2—C16—H16	108.9
C5—C4—C3	116.23 (15)	C17—C16—H16	108.9
C5—C4—H4	121.9	N2—C16—H16B	108.9
C3—C4—H4	121.9	C17—C16—H16B	108.9
C4—C5—C6	122.05 (16)	H16—C16—H16B	107.7
C4—C5—H5	119.0	C22—C17—C18	118.45 (16)
C6—C5—H5	119.0	C22—C17—C16	117.98 (14)
C7—C6—C5	121.71 (16)	C18—C17—C16	123.51 (14)
C7—C6—H6	119.1	C17—C18—C19	120.39 (18)
C5—C6—H6	119.1	C17—C18—H18	119.8
C6—C7—C8	116.38 (15)	C19—C18—H18	119.8
C6—C7—H7	121.8	C20—C19—C18	120.4 (2)
C8—C7—H7	121.8	C20—C19—H19	119.8
C7—C8—C3	122.05 (14)	C18—C19—H19	119.8
C7—C8—N2	131.20 (14)	C19—C20—C21	119.68 (19)
C3—C8—N2	106.69 (12)	C19—C20—H20	120.2
N1—C9—C10	111.98 (12)	C21—C20—H20	120.2
N1—C9—H9	109.2	C20—C21—C22	120.6 (2)
C10—C9—H9	109.2	C20—C21—H21	119.7
N1—C9—H9B	109.2	C22—C21—H21	119.7
C10—C9—H9B	109.2	C21—C22—C17	120.4 (2)
H9—C9—H9B	107.9	C21—C22—H22	119.8
C15—C10—C11	118.51 (16)	C17—C22—H22	119.8
C15—C10—C9	121.09 (15)		
			
C8—N2—C2—N1	0.33 (16)	C16—N2—C8—C3	−175.22 (12)
C16—N2—C2—N1	175.59 (12)	C2—N1—C9—C10	−93.34 (17)
C8—N2—C2—C1	−179.08 (14)	C3—N1—C9—C10	86.67 (17)
C16—N2—C2—C1	−3.8 (2)	N1—C9—C10—C15	−93.28 (17)
C3—N1—C2—N2	−0.73 (16)	N1—C9—C10—C11	88.23 (18)
C9—N1—C2—N2	179.28 (13)	C15—C10—C11—C12	0.7 (3)
C3—N1—C2—C1	178.67 (14)	C9—C10—C11—C12	179.22 (19)
C9—N1—C2—C1	−1.3 (2)	C10—C11—C12—C13	0.4 (4)
C2—N1—C3—C8	0.84 (15)	C11—C12—C13—C14	−0.8 (4)
C9—N1—C3—C8	−179.17 (12)	C12—C13—C14—C15	0.0 (4)
C2—N1—C3—C4	−175.93 (15)	C11—C10—C15—C14	−1.5 (3)
C9—N1—C3—C4	4.1 (2)	C9—C10—C15—C14	−179.97 (16)
C8—C3—C4—C5	1.1 (2)	C13—C14—C15—C10	1.1 (3)
N1—C3—C4—C5	177.47 (15)	C2—N2—C16—C17	100.24 (17)
C3—C4—C5—C6	−0.5 (2)	C8—N2—C16—C17	−85.18 (17)
C4—C5—C6—C7	−0.3 (3)	N2—C16—C17—C22	152.21 (15)
C5—C6—C7—C8	0.5 (3)	N2—C16—C17—C18	−30.7 (2)
C6—C7—C8—C3	0.1 (2)	C22—C17—C18—C19	0.4 (3)
C6—C7—C8—N2	−176.73 (15)	C16—C17—C18—C19	−176.69 (16)
C4—C3—C8—C7	−1.0 (2)	C17—C18—C19—C20	−0.3 (3)
N1—C3—C8—C7	−178.13 (13)	C18—C19—C20—C21	0.3 (4)
C4—C3—C8—N2	176.55 (13)	C19—C20—C21—C22	−0.3 (4)
N1—C3—C8—N2	−0.62 (14)	C20—C21—C22—C17	0.3 (3)
C2—N2—C8—C7	177.39 (15)	C18—C17—C22—C21	−0.4 (3)
C16—N2—C8—C7	2.0 (2)	C16—C17—C22—C21	176.83 (18)
C2—N2—C8—C3	0.20 (15)		
